# Surgical treatment of interstitial pregnancy without cornual resection: A case report

**DOI:** 10.1097/MD.0000000000029730

**Published:** 2022-06-30

**Authors:** Qian Feng, Jie Zhong, Yujie Liu, Shu-Ting Li, Lili Zong

**Affiliations:** a Department of Obstetrics and Gynaecology, Monash University, Melbourne, Australia; b Department of Gynaecology, the First Affiliated Hospital of Guangzhou University of Chinese Medicine, Guangzhou, China; c Department of Medical Imaging, the First Affiliated Hospital of Guangzhou University of Chinese Medicine, Guangzhou, China.

**Keywords:** cornual pregnancy, ectopic pregnancy, interstitial pregnancy

## Abstract

Interstitial pregnancy (IP) is a rare but extremely life-threatening form of ectopic pregnancy. The traditional surgical treatment for this anomaly is the resection of uterine cornua or fallopian tubes, which often damages their structural integrity, thereby compromising the reproductive potential for women who wish to preserve fertility.

**Patient concerns:**

A 33-year-old female was admitted to our hospital with suspected ectopic pregnancy, following a 4-weeks history of positive pregnancy tests after uterine evacuation. The patient was hemodynamically stable on arrival. Ultrasound revealed an empty uterus with an eccentric gestational sac located at the fundus and surrounded by a thin myometrium, indicative of a suspected interstitial pregnancy.

**Diagnosis and intervention:**

After failed attempt at medical management with a single dose of intramuscular methotrexate, the patient was arranged for hysteroscopy-assisted laparoscopy. In surgery, the uterine cavity appeared empty, and a 2 × 2 cm bulge with increased vascularity at the right uterine courna was identified upon examination. The gestational sac was aspirated through the vagina from the right ostium of the uterine tube using a suction curette pointing at the right ostium. Sutures were not needed afterward, and the myometrial anatomy was left undisrupted. The diagnosis of IP was confirmed by the postoperative histological report.

**Outcomes:**

Perioperative blood loss was approximately 10 ml and the operative time was 40 minutes. The patient had an uneventful postoperative recovery and was discharged after 3 days. Subsequent follow-ups showed a significant reduction in the patient serum beta hCG to 48IU/L within 5 days postoperation, and a negative result after 7 days.

**Lessons:**

This novel surgical technique is an alternative minimally-invasive approach for selected early diagnosed and hemodynamically stable IP patients. The technique represents a safe, quick, and simple approach combining the benefits of laparoscopy, such as allowing for immediate conversion of cornuectomy when uterus ruptures, and the benefits of suction curettage, such as shorter operative time and minimal blood loss. We believe patients with interstitial pregnancy who still have fertility wishes would benefit from this surgical technique to a larger extent in the future.

## 1. Introduction

Interstitial pregnancy (IP) is a rare type of ectopic pregnancy, defined as zygote implantation within the proximal intramural portion of the fallopian tubes. Given the abundant blood supply and poor extensibility at this region, the continued growth of the gestational sac may lead to a catastrophic hemorrhage. This explains why the mortality rate observed in IP is 7 times higher than ectopic pregnancy in general, accounting for one-fifth of all deaths associated with ectopic pregnancies.^[1,2]^ Traditional surgical intervention of IP includes cornuectomy and exploratory laparotomy, which are associated with long intraoperative time and a high risk of massive hemorrhage,^[3]^ possibly necessitating a hysterectomy and leaving the women with irreversible infertility. With the advancement in surgical techniques, these cases have been managed safely using novel and minimally-invasive approaches.^[4,5]^ Herein, we describe a novel surgical technique for managing IP in a fertility-sparing manner without disrupting the myometrial architecture.

## 2. Case presentation

A 33-year-old, gravida 5, para 2, female presenting with 9 weeks of amenorrhea was admitted to our hospital on November 16, 2016. Four weeks before the admission, she had an abortion by vacuum aspiration at a local hospital for an unwanted pregnancy; however, her serum β human chorionic gonadotropin (β-hCG) levels remained high 2 weeks after the surgery, and that prompted another uterine evacuation in the same hospital for suspected retained products of conception. Positive pregnancy test results persisted even after the repeated surgical procedure.

On arrival, the patient had no other discomforts apart from slight nausea. She did not complain of any abdominal pain or vaginal bleeding. On vaginal examination, there was no abdominal wall tenderness, and the cervical OS was closed without bleeding. The uterus was retroverted, parous size, without fullness or tenderness in the fornix. She has had 2 vaginal deliveries and expressed desires for future pregnancy.

On laboratory examination, the only abnormal finding was the patient’s high serum β-hCG level, which was at 27,184 IU/L. Transvaginal pelvic ultrasound revealed an empty uterus measuring 65 × 58 × 42 mm and a 27 × 17 × 10 mm mass located eccentrically on the right uterine fundus surrounded by a myometrial margin of only 4.8 mm thin. An “interstitial line sign,” an echogenic line extending from the mass to the endometrial stripe, was also seen. No free fluid was identified in the cul-de-sac. In view of the high levels of serum β-HCG and ultrasound findings, a diagnosis of IP was suggested. Her willingness to preserve fertility prompted the administration of a single dose of methotrexate (MTX) (50 mg/m^2^) intramuscularly. However, 3 days after the administration of the MTX, her serum β-hCG level remained almost unchanged, at 26,927 IU/L. After counseling for possible treatment modalities, the decision to perform laparoscopy-assisted hysteroscopy was made.

**Figure 1.**
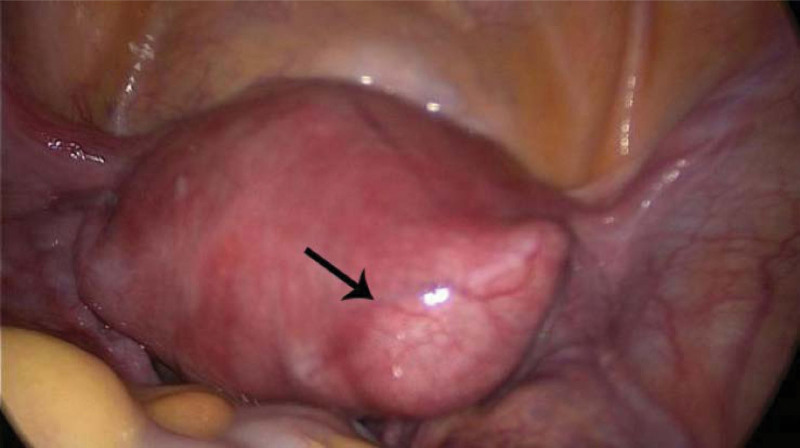
The initial view of the uterus at laparoscopy (the black arrow denotes the bulge with increased vascularity at the right cornua).

**Figure 2.**
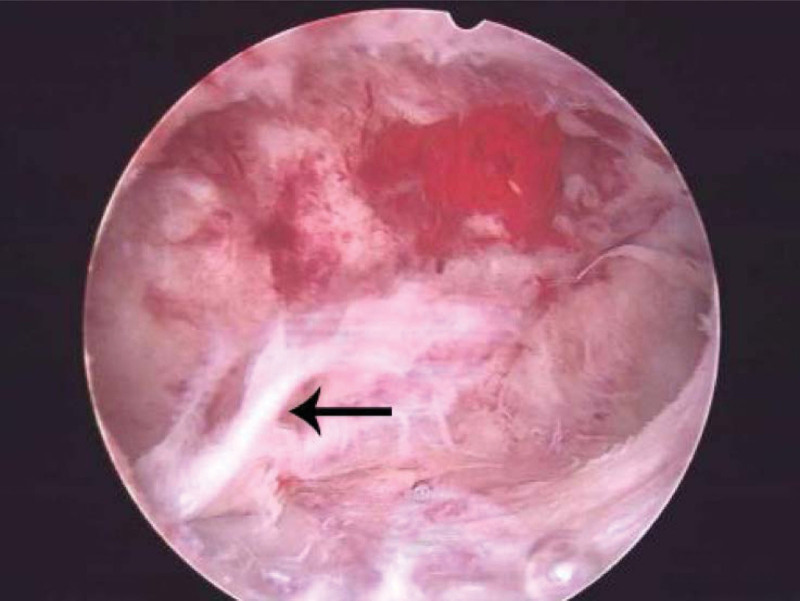
Villus-like tissue (the black arrow) floating at the right ostium under hysteroscopy.

**Figure 3.**
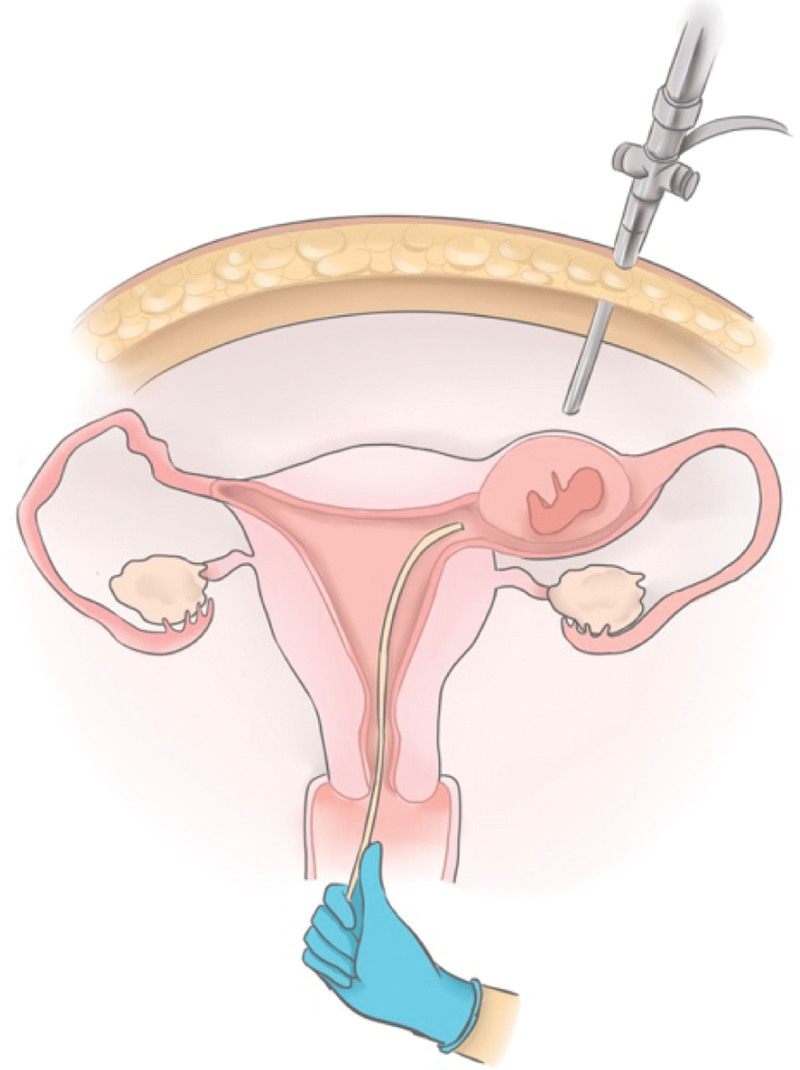
The graphic illustration of transvaginal gestational sac aspiration under laparoscopy guidance.

**Figure 4.**
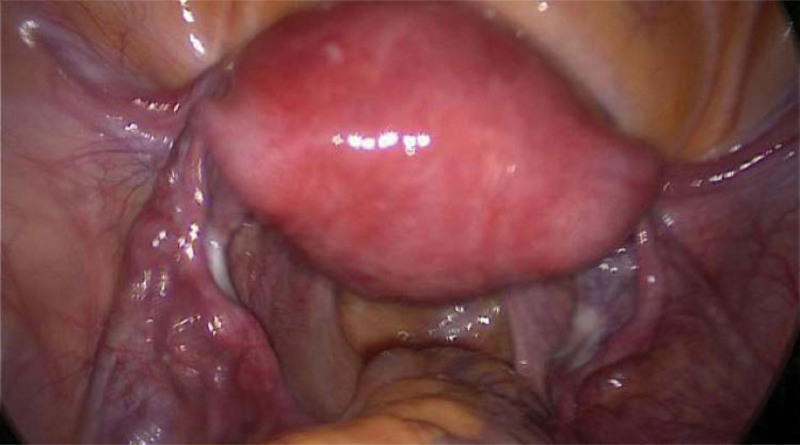
The bulge at the cornual region disappeared immediately after the tissue was aspirated.

During the laparoscopy, we observed a 20 × 20 mm bulge with increased vascularity located in close proximity to the insertion of the round ligament into the uterus (Fig. 1). After confirming there was no active pelvic bleeding, we then introduced the hysteroscope with a water pressure of 80 mm Hg. The hysteroscopy showed an empty uterus with the right ostium obstructed by a white villus-like tissue while the left was open (Fig. 2). We suspected the floating tissue was a part of the ectopic gestational sac, with the remaining part concealed within the fallopian tube. To remove the gestational sac, we introduced a suction curette with a 6 mm diameter into the uterus, advanced and rotated with its tip placed at the right tubal ostium, where the gestational sac was concealed (Fig. 3). Under laparoscopic supervision, transvaginal aspiration of the gestational sac was carried out. While the ectopic gestational sac was being evacuated, cornual thickening was noticed to be shrinking simultaneously (Fig. 4). No further bleeding was found when the hysteroscope was introduced again. The total operating time was noted to be 40 minutes with an estimated 10 ml blood loss. The patient was discharged on postoperative day 3. Her serum β-hCG levels progressively decreased to 48 IU/L 5 days after the surgery; subsequently, her urine pregnancy test became negative after a week. The histological features were consistent with the diagnosis of interstitial pregnancy.

## 3. Discussion

Despite the fact that interstitial pregnancy is one of the fatal forms of ectopic pregnancy, there exists no consensus about its management due to its rarity. Conservative treatment of these cases includes MTX injection, administered systemically or locally in the uterus; however, this approach is associated with a high rate of failure, ranging from 9% to 65%.^[6]^ When the serum β-HCG exceeds 20,000 IU/L, poor response to MTX is highly likely and may warrant further surgical treatment; this is consistent with our case.^[7]^ The widely used surgical intervention for IP is cornual wedge resection. The major disadvantage of this treatment is disruption of myometrial lining and therefore impairs fertility potential. By contrast, novel fertility-sparing and minimally-invasive surgical interventions, such as hysteroscopy assisted by ultrasound and uterine evacuation under laparoscopic guidance, have been shown to be safe and effective for treating women with IP.^[6,8]^ These new techniques not only represent a strength allowing for the preservation of the uterine lining for future pregnancy, but they also have a short surgery time and minimal blood loss. A case synonymous with our case was reported by Zhang et al, who performed laparoscopy-assisted transvaginal suction curettage in 3 women with IP; the average intraoperative blood loss was below 50 ml and the surgery duration was shorter than 20 minutes.^[9]^

In contrast to our case, using laparoscopy to guide the procedure of transvaginal gestational sac aspiration, existing studies by other authors often employed transabdominal ultrasound instead.^[10,11]^ We chose laparoscopy because they are more likely to pick up bleeding points and allow immediate bleeding control when abrupt hemorrhage occurs. Regarding the instrument used to evacuate the gestational sac, published case reports used various and innovative instruments that are “borrowed” from other specialties, such as urology and pediatrics. These instruments include vacuum curette, suction cannula, 8 F flexible pediatric suction catheter, and Novy™ Cornual and Cannulation Set (Cook Medical, Bloomington, IN).^[6,8]^ This variety in instruments demonstrated the importance of multidisciplinary collaboration when managing rare and challenging cases; however, they should be used with caution and awareness of possible uterine injury. There have been at least 2 cases of uterine perforations in women undergoing transvaginal aspiration using an 8 F flexible pediatric suction catheter, all of which were immediately converted to cornual resection.^[8]^

It should also be noted that the surgical technique presented in our case can only be performed in the selected population within an appropriate clinical context. Specifically, they should be early diagnosed and hemodynamically stable, and they should show no sign of uterine ruptures such as progressive abdominal pain or increased free fluid in the cul-de-sac. Preferably, their ectopic gestational sac should be located in proximity to the ostium, giving the surgical instruments easy accessibility. Furthermore, the undertaking of this technique requires the presence of a skilled surgeon; one who is able to perform corneal resection or hysterectomy and can manage hemorrhage as and when they occur.

## 4. Conclusions

To conclude, the transvaginal aspiration of the ectopic gestational sac in selected women with interstitial pregnancy is a quick, safe, and minimally-invasive surgical technique. We recommend that clinicians consider this technique before cornual wedge resection for patients who are hemodynamically stable and in the presence of a proficient surgeon. More fertility-desiring women would benefit from its application on a larger scale in the future.

## Acknowledgment

We would like to thank Emmanuel Patrick Dumfeh from Hainan Medical University for English language editing.

## Author contributions

Conceptualization: Qian Feng and Lili Zong.

Methodology: Jie Zong and Yujie Liu.

Writing – original draft: Qian Feng.

Writing – review & editing: Qian Feng, Jie Zong, Yujie Liu, Shuting Li, and Lili Zong.
